# A Biochemometric Approach for the Identification of In Vitro Anti-Inflammatory Constituents in Masterwort 

**DOI:** 10.3390/biom10050679

**Published:** 2020-04-28

**Authors:** Julia Zwirchmayr, Ulrike Grienke, Scarlet Hummelbrunner, Jacqueline Seigner, Rainer de Martin, Verena M. Dirsch, Judith M. Rollinger

**Affiliations:** 1 Department of Pharmacognosy, Faculty of Life Sciences, University of Vienna, Althanstraße 14, 1090 Vienna, Austria; julia.zwirchmayr@univie.ac.at (J.Z.); ulrike.grienke@univie.ac.at (U.G.); scarlet.hummelbrunner@univie.ac.at (S.H.); verena.dirsch@univie.ac.at (V.M.D.); 2 Department of Vascular Biology and Thrombosis Research, Medical University of Vienna, Schwarzspanierstaße. 17, 1090 Vienna, Austria; jacqueline.seigner@meduniwien.ac.at (J.S.); rainer.demartin@meduniwien.ac.at (R.d.M.)

**Keywords:** *Peucedanum ostruthium*, Apiaceae, ELINA, HetCA, STOCSY, coumarines, NF-ĸB, VCAM-1, E-selectin, inflammation

## Abstract

*Peucedanum ostruthium* (L.) Koch, commonly known as masterwort, has a longstanding history as herbal remedy in the Alpine region of Austria, where the roots and rhizomes are traditionally used to treat disorders of the gastrointestinal and respiratory tract. Based on a significant NF-κB inhibitory activity of a *P. ostruthium* extract (PO-E), this study aimed to decipher those constituents contributing to the observed activity using a recently developed biochemometric approach named ELINA (Eliciting Nature’s Activities). This -omics tool relies on a deconvolution of the multicomponent mixture, which was employed by generating microfractions with quantitative variances of constituents over several consecutive fractions. Using an optimized and single high-performance counter-current chromatographic (HPCCC) fractionation step 31 microfractions of PO-E were obtained. ^1^H NMR data and bioactivity data from three in vitro cell-based assays, i.e., an NF-ĸB reporter-gene assay and two NF-κB target-gene assays (addressing the endothelial adhesion molecules E-selectin and VCAM-1) were collected for all microfractions. Applying heterocovariance analyses (HetCA) and statistical total correlation spectroscopy (STOCSY), quantitative variances of ^1^H NMR signals of neighboring fractions and their bioactivities were correlated. This revealed distinct chemical features crucial for the observed activities. Complemented by LC-MS-CAD data this biochemometric approach differentiated between active and inactive constituents of the complex mixture, which was confirmed by NF-κB reporter-gene testing of the isolates. In this way, four furanocoumarins (imperatorin, ostruthol, saxalin, and 2’-O-acetyloxypeucedanin), one coumarin (ostruthin), and one chromone (peucenin) were identified as NF-κB inhibiting constituents of PO-E contributing to the observed NF-ĸB inhibitory activity. Additionally, this approach also enabled the disclose of synergistic effects of the PO-E metabolites imperatorin and peucenin. In sum, prior to any isolation an early identification of even minor active constituents, e.g. peucenin and saxalin, ELINA enables the targeted isolation of bioactive constituents and, thus, to effectively accelerate the NP-based drug discovery process.

## 1. Introduction

Natural products (NPs) have been used for millennia as part of herbal remedies to alleviate and treat various types of diseases. Their unique chemical diversity provides a vast source of drug-like molecules endowed with various biological activities, which account for their significant contributions in drug discovery [1,2,3,4]. Until now, anti-inflammatory activities of NPs are one of the most frequently reported effects and have been described for many traditionally used herbal drugs in vitro and partly also in vivo, e.g., from *Zingiber officinale* Roscoe [5,6,7], *Symphytum officinale* L. [8,9], *Vaccinium myrtillus* L. [10,11,12], and *Calendula officinalis* L. [13,14], as well as for constituents of herbal remedies, such as curcumin [15,16,17,18,19] and resveratrol [20,21,22]. However, because of the intrinsic complexity of the inflammation process, the search for new anti-inflammatory compounds remains a challenging task [23]. Inflammation underlies a wide range of physiological and pathological changes and is triggered by microbial, chemical or physical stimuli, such as infection, tissue injury, and traumata. Activation of NF-ĸB leads to an enhanced expression of the cell adhesion molecules ICAM-1, VCAM-1, and E-selectin. Here, the activation of endothelial cells in blood vessels near the site of the injury plays a crucial role during the inflammation cascade as it promotes the chemoattraction, adhesion, and transmigration of leucocytes in the affected tissue. Hence, NF-κB plays a central role in the transcriptional regulation of inflammatory mediators and represents a rational target for intervention [8,24,25]. 

Nature is a prolific source of novel secondary metabolites, able to interfere with key players in inflammation [23] and their multi-target properties play an advantageous role when dealing with complex diseases. The structural diversity of NPs allows for identifying novel bioactives or the recognition of similar congeners with potential activity [26]. A previously published ethnopharmacological study on 71 Austrian herbal drugs, traditionally used to treat inflammation, revealed NF-κB inhibition for a detannified methanol extract and a dichloromethane extract generated from the roots of *Peucedanum ostruthium* (L.) W. D. J. Koch, also known as masterwort [27]. Masterwort, belonging to the Apiaceae family, has a longstanding history as a herbal remedy in the Alpine region of Austria. The rhizomes and roots (Radix Imperatoriae) are traditionally used to treat disorders of the respiratory tract, the cardiovascular system [28] as well as gastrointestinal diseases like stomach pain or ulcer [29]. One main compound class present in *P. ostruthium* are coumarins, such as osthole and ostruthin, as well as furanocoumarins such as oxypeucedanin, ostruthol, or imperatorin [28]. Although several studies on the isolation and identification of coumarins from *P. ostruthium* have been performed, the secondary metabolites responsible for the observed NF-ĸB inhibition of masterwort root extracts remain elusive. 

One goal in NP-based drug discovery is to disclose constituents from complex mixtures responsible for a certain biological effect. Traditionally, bioassay-guided fractionation is applied as the gold standard to simplify these complex mixtures (i.e., extracts) and to isolate the comprised bioactive constituents. Although this approach has led to the discovery of drugs with great significance (e.g., taxol and artemisinin) [30], bioassay-guided fractionation has its restrictions: (i) bioactive compounds in low quantity are easily overlooked because of the presence of highly abundant compounds, (ii) bioactivity can get lost due to degradation or adhesion of compounds to chromatographic materials during repetitive fractionation steps [31], and (iii) synergistic or additive effects among several compounds are difficult to recognize [1,30,32,33,34]. Due to its intrinsic complexity, NP research is hampered by tedious fractionation and isolation steps that often results in the repeated isolation and identification of already known constituents. This can be avoided by the early identification of known NPs in complex mixtures (i.e., dereplication) [35]. In the recent past, there has been a great interest in the implementation of bioactivity data with chemical profiles to unveil the biologically active constituent(s) in multicomponent mixtures [33,36,37]. This so-called biochemometric approach is achieved by the correlation of biological and chemical datasets via multivariate statistics [33,36,37], as recently implemented by the NMR- and MS-based ELINA approach (Eliciting Nature’s Activities) [32]. ELINA offers significant benefits over bioassay-guided fractionation, namely (i) the semi-quantitative estimation of secondary metabolite levels in mixtures by LC hyphenated to MS and a charged aerosol detector (CAD) [35]; (ii) the quantitative composition of secondary metabolites via ^1^H NMR, and (iii) the in situ structural characterization of bioactives and inactive constituents prior to isolation by statistically correlating ^1^H NMR profiles to bioactivity data [36,38]. Whereas in previous similar biochemometric approaches, structural data were correlated with bioactivity data derived from enzyme assays [36,37,38], we here probed the robustness and predictive power of ELINA when using the readout from a cell-based assay. This application study accordingly aimed to disclose those metabolites in the extract of masterwort, which contributes to its previously found NF-ĸB inhibitory activity. To achieve this aim, we applied the following workflow:

(i) microfractionation of a bioactive extract prepared from the roots of *P. ostruthium* using high-performance counter-current chromatography (HPCCC);
(ii) investigation of the masterwort extract and its generated microfractions for their ability to interfere with the NF-ĸB signaling pathway, and thus the expression of pro-inflammatory target genes (E-selectin, VCAM-1) in cell-based models;
(iii) recording of ^1^H NMR and LC-MS-CAD data of all microfractions;
(iv) correlation of structural data with bioactivity data to structurally identify and distinguish the bioactive/inactive metabolites from the extract, and
(v) isolation and assaying of ELINA predicted active and inactive constituents from the masterwort root extract for validation of the used approach.

## 2. Materials and Methods 

### 2.1. General Experimental Procedures

HPCCC fractionation was performed on a Spectrum HPCCC instrument (Dynamic Extractions Ltd) connected with an isocratic solvent pump (ecom Alpha 10+), a fraction collector and a chiller. UPLC analysis was performed on a Waters Acquity UPLC system (H-class) equipped with a binary solvent manager, a sample manager, a column manager, a PDA detector, an ELSD, and a fraction collector using a Waters Acquity UPLC BEH Phenyl column (1.7 μm, 2.1 × 100 mm). PO01_01-PO01_31 were chromatographed over a Dionex HPLC connected to a charged aerosol detector (CAD) and an MS Iontrap (LTQ XL™ Linear Ion Trap Mass Spectrometer, Thermo Fisher Scientific Inc. Bremen, Germany) equipped with ESI with a Waters Acquity UPLC BEH Phenyl column (1.7 μm, 2.1 × 100 mm). Flash chromatography was performed on an Interchim puriFlash 4250 system (Montluçon, France), equipped with an evaporative light scattering detector (ELSD), a photodiode array (PDA) detector, and a fraction collector, controlled by Interchim Software. Sephadex column chromatography (CC) was performed with Sephadex LH-20 in 100% MeOH. TLC analyses were performed with toluol:ether:10% aqueous acetic acid (1:1:1, upper layer) as mobile phase. Stationary phase: Merck silica gel 60 PF254, detection under both visible light and UV254 and UV366. NMR experiments were performed on a Bruker Avance 500 NMR spectrometer (UltraShield) (Bruker, Billerica, MA) with a 5 mm probe (TCI Prodigy CryoProbe, 5 mm, triple resonance inverse detection probe head) with z-axis gradients and automatic tuning and matching accessory (Bruker BioSpin). The resonance frequency for ^1^H NMR was 500.13 MHz and for ^13^C NMR 125.75 MHz. Standard 1D and gradient-enhanced (ge) 2D experiments, like double quantum filtered (DQF) COSY, HSQC, and HMBC, were used as supplied by the manufacturer. (Ultrahigh-)gradient grade solvents from Merck (Darmstadt, Germany) and deuterated solvents from Deutero GmbH (Kastellaun, Germany) were used. 

### 2.2. Plant Material

Dried *Peucedanum ostruthium* roots and rhizomes were purchased from Kottas Pharma GmbH (Ch.Nr.: P17301770), Vienna. A voucher specimen (JR-20180119-A2) is deposited at the Department of Pharmacognosy, University of Vienna, Austria.

### 2.3. Extraction 

For the extraction of *P. ostruthium* the protocol of [39,40] was used and modified as follows: 1 kg dried plant material was defatted with 2 L *n*-hexane (*n*-hex). The flasks were shaken for three days. The obtained *n*-hex extract was discarded. The remaining defatted plant material was extracted with 2.6 L CH_2_Cl_2_ and shaken for two days. The CH_2_Cl_2_ extract was transferred into a round bottom flask and the solvent was evaporated on a rotary evaporator. The remaining plant material was extracted with 2.6 L CH_2_Cl_2_ for a second time and shaken for one day. All CH_2_Cl_2_ extracts were combined and dried. The remaining plant material was extracted with 2.6 L MeOH for 7 days and filtrated. For an exhaustive extraction, this procedure was repeated twice. CH_2_Cl_2_/MeOH extracts were combined and concentrated to dryness on a rotary evaporator. The extraction yielded 348.96 g extract (PO-E; 35.81%). 

### 2.4. UPLC Analysis of PO-E

PO-E was chromatographed over UPLC using a binary mobile phase system consisting of A) H_2_O and B) CH_3_CN. The gradient was from 13%–98% B in 12 min followed by 5 min re-equilibration. Method in detail: 13% B for 0.5 min, 13%–18% B in 0.5 min, 18%–45% B in 1 min, isocratic 45% B for 1.7 min, 45%–73% B in 2.8 min, 73%–98% B in 0.3 min, isocratic 98% B for 5 min, 98%–13% B in 0.1 min, isocratic 13% B for 0.1 min; Conditions: temperature, 40 °C; flow rate, 0.300 mL/min; injection volume, 1 μL. Detection of compounds using PDA and ELSD. PDA conditions: 210 nm and full range spectra 192–400 nm. 

### 2.5. HPCCC Separation Procedure

#### 2.5.1. Selection of Two-phase Solvent System for HPCCC

Mixtures of *n*-hex, ethyl acetate (EtOAc), methanol (MeOH), and H_2_O (HEMWat) with various volume ratios were used for the two-phase solvent system selection. Briefly, a small amount of selected HEMWat solvent mixtures was prepared in small test-tubes with ground joint, whereas one aliquot of the MeOH ratio was replaced by an aliquot of a sample solution of PO-E (5 mg/mL in MeOH). The mixture was shaken vigorously followed by a 10 min equilibration time at room temperature. 1.00 mL of each phase was taken, dried and re-dissolved in 500 *µ*L MeOH for UHPLC analysis. To find the ideal solvent system(s) for the micro-fractionation, the partition coefficient K_D_ was determined for each solvent system, as described by Garrard [41]: This was done for both, reversed-phase and normal-phase mode. Partition coefficients were expressed as the peak area of selected peaks in the stationary phase divided by the peak area of the corresponding peak in the mobile phase (data not shown). HEMWat systems resulting in K_D_ values for the main constituents in the range of 0.5 to 5.0 (i.e., systems 22, 21, 19, 17, 15, and 10) were selected for the semi-preparative HPCCC analysis.

#### 2.5.2. Microfractionation with HPCCC

Microfractionation of PO-E was performed in a semi-preparative, normal-phase mode with gradient elution, starting with HEMWat system 22 and gradually increasing the polarity of the mobile phases by subsequently applying the mobile phases (i.e., upper layer) of HEMWat system 21, 20, 19, 17, 15, and 10 (Supporting Information, Table S1). The semi-preparative column was initially filled with the stationary phase (i.e., lower layer) of HEMWat system 22 at 200 rpm with a flow rate of 10 mL/min. After rotating up to 1600 rpm the mobile phase of HEMWat system 22 was pumped through the column with a flow rate of 6.0 mL/min. After the equilibrium was reached, the sample solution of PO-E (269.82 mg dissolved in 10 mL of HEMWat system 22) was injected and the fraction manager was set to 1 min time count (i.e., 6 mL/fraction). For each solvent system, 250 mL of the upper layer were used as the mobile phase for the fractionation, starting with 250 mL of the mobile phase of system 22. When the volume of system 22 was reduced to 60 mL, 50 mL of the upper layer of system 21 were added on top. As the solvent mixture of system 22 and 21 was reduced to 60 mL the remaining 200 mL of the upper layer of system 21 were added. This procedure was repeated for each selected solvent system, resulting in an even fractionation. Elution extrusion was performed with the lower, stationary phase of system 10 at 200 rpm and 10 mL/min. 285 fractions were collected. A second run with 329.40 mg was performed, resulting in 280 fractions. All HPCCC fractions were monitored by TLC and pooled to obtain 31 final microfractions, i.e., PO01_01–PO01_31. (Table S2, Figure S1).

### 2.6. NMR Measurements of PO01_01-PO01_31 

The samples were measured at 298 K in fully deuterated methanol referenced to the residual non-deuterated solvent signal at 3.31 ppm (MeOH). Dry weighted samples (between 2.3 and 2.4 mg) of PO01_01-PO01_31 were dissolved in methanol-d4 to reach a concentration of 3.11 mg/mL. To avoid precipitation in the NMR tube, an aliquot of 750 μL of each fraction was put into an Eppendorf tube and centrifuged at 3000 rpm for 5 min. From the supernatants, 645 μL were transferred to NMR tubes. 

Standard ^1^H NMR spectra with 16 scans and relaxation delay of 6 s were recorded for all fractions using pulse sequences included in the standard pulse program library of Bruker. TopSpin 4.0, controlling a 60-position autosampler, was used for fully automated NMR operation, i.e., temperature control, sample loading, tuning and matching, shimming, lock phase optimization, 90° pulse calibration, and data recording. For the pure compounds, both standard 1D and 2D experiments were performed.

### 2.7. LC-MS-CAD Measurements of PO01_01–PO01_31 

PO01_01-PO01_31 (c = 2 mg/mL in MeOH) were chromatographed using a binary mobile phase system consisting of A) H_2_O: formic acid (100:0.01) and B) CH_3_CN. The gradient was from 13%–98% B in 20 min. Isocratic 13% B for 0.5 min, 13%–18% B in 0.5 min, 18%–45% B in 1 min, isocratic 45% B for 1.7 min, 45%–73% B in 2.8 min, 73%–98% B in 0.3 min, isocratic 98% B for 5.2 min, 98%–13% B in 0.5 min, isocratic 13% B for 7.5 min; conditions: temperature, 40 °C; flow rate, 0.300 mL/min; injection volume, 10 μL. Detection of compounds using PDA and CAD. PDA conditions: 210 nm and full range spectra 192–400 nm. CAD nebulizer temperature, 35 °C. Mass conditions: source heater temperature, 300 °C; source voltage, 3.7 kV; sheath gas flow rate, 40; aux gas flow rate, 10.

### 2.8. ^1^H NMR Spectra Processing and Statistical Correlation with Bioactivity Data

For spectral alignment, all ^1^H NMR spectra of the 31 microfractions were subjected to chemical shift scale calibration by referencing to the MeOH resonance at 3.31 ppm. Then, a baseline correction factor was applied using a simple polynomial curve fitting of the mathematical equation A + Bx + Cx2 + Dx3 + Ex4. Baseline correction was carried out manually using the appropriate factors. To detect structural features of the active components prior to any purification, the previously described heterocovariance (HetCA) analysis was applied [36]. Briefly, ^1^H NMR spectra of relevant fraction packages were bucketed (covered range: *δ*_H_ 0.5–10; bucket width: 0.0005 ppm). This means that spectra were reduced in their complexity by summation of all the data points per bucket. Since each bucket width was 0.0005 ppm, this procedure gave a total of 19,000 spectroscopic buckets. The intensities of ^1^H NMR resonances of each bucket were calculated and served as variables for subsequent analyses. Covariance as a measure of the joint variability between the two variables (i) ^1^H NMR resonance intensity and (ii) percentage of NF-ĸB inhibition at 10 μg/mL was calculated. Additionally, the normalized version of covariance, i.e., the correlation coefficient, was calculated for color coding. Thus, the resulting bucket-specific covariance values were plotted as ^1^H NMR pseudo-spectrum and color coded according to the respective correlation coefficients. This procedure allowed for the straightforward identification of ^1^H NMR resonances which are either positively (red) or negatively (blue) correlated with NF-κB inhibition. HetCA analysis was carried out with spectra of selected sets of fractions which showed a distinct variation in bioactivity and concentration of contained secondary metabolites. Additionally, the statistical total correlation spectroscopy (STOCSY) was applied [42], using the multicolinearity of the intensity variables over a set of spectra to give the correlation among the intensities of the various resonances across the whole set of spectra. STOCSY displays also covariance as a function of spectroscopic position and is color-coded according to the respective correlation coefficients (i.e., the intensities of the various resonances across the whole fraction package). STOCSY allows for the detection of multiple ^1^H NMR signals from the same molecule based on the multi-collinearity of their intensities in the selected set of ^1^H NMR spectra. The calculations for HetCA and STOCSY analyses were performed using the multi-paradigm numerical computing environment MATLAB.

### 2.9. Targeted Isolation of Bioactives

For the isolation of peucenin (**2**), PO-E was fractionated via isocratic HPCCC in semi-preparative normal-phase mode, using an optimized HEMWat system consisting of *n*-hex:EtOAc:MeOH:H_2_O (volume ratios 1.8:1.2:2.1:0.9). The sample solution of PO-E (310 mg dissolved in 10 mL of optimized HEMWat system) was injected and fractionation was performed at 1600 rpm with a flow rate of 6.0 mL/min. The fraction manager was set to 1 min time count (i.e., 6 mL/fraction). In total, 79 tubes were collected, monitored with TLC and compared with the TLC pattern of the active microfraction PO01_16. Tubes 42–49 were pooled (5.38 mg) and purified via Sephadex CC collecting 60 sub-fractions. Sub-fractions 31–50 were combined yielding 3.12 mg of **2**. Fraction PO01_22 (3.33 mg) was fractionated via Sephadex CC yielding 79 tubes. Saxalin (**4**) (1.06 mg) was isolated by pooling fraction 46–57. Ostruthol (**5**) and oxypeucedanin methanolate (**6**) were isolated by combining PO01_24 and PO01_25 (~19 mg). The combined microfractions were separated via Sephadex CC, yielding five fractions PO02_01-PO02_05. PO02_02 (3.83 mg), PO02_03 (3.47 mg), and PO02_04 (5.31 mg) were further fractionated with flash chromatography via direct injection (PuriFlash C_18_ HQ column (6 g); flow rate: 2 mL/min) applying a gradient system of CH_3_CN/water as mobile phase (0 min 40%/60%, 3 min 40%/60%, 13 min 60%/40%, 16 min 98%/2%, 20 min 98%/2%, 20 min 40%/60%, 23 min 40%/60%). 3 × 40 tubes were collected and chromatographed with TLC. Tubes were pooled according to their TLC pattern yielding five fractions PO03_01–PO03_05. Fractions PO03_01, PO03_03, and PO03_05 were combined, yielding 4.27 mg of **5**. Fractions PO03_02 and PO03_04 were combined, yielding 1.05 mg of **6**.

The purity of all isolated compounds was determined by UPLC-ELSD analysis to be >98%. 

### 2.10. Cell-Lines, Chemicals and Biochemicals 

#### 2.10.1. Reporter-Gene Assay 

HEK293 cells stably transfected with the NF-κB-driven luciferase reporter gene NF-κB-luc (293/NF-κB-luc cells, Panomics, RC0014) were stained with 2 µM cell tracker green (CTG, Thermo Scientific). After one hour, 4 × 10⁴ cells per well were seeded in a 96 well plate in serum-free DMEM (4.5 g/L Glucose) obtained from Lonza and supplemented with 2 mM glutamine, 100 U/mL benzylpenicillin and 100 µg/mL streptomycin. After incubation at 37 °C, 5% CO_2_ overnight, the cells were pre-treated on the next day with the samples for 1 h. Thereafter, cells were stimulated with 2 ng/mL human recombinant TNF-α (Sigma) for 3.5 h to activate the NF-κB signaling pathway. Then the medium was removed and cells were lysed with luciferase reporter lysis buffer (E3971, Promega, Madison, USA). PO-E and its microfractions were tested at a concentration of 10 µg/mL in at least three independent experiments, if not otherwise indicated. The sesquiterpene lactone parthenolide, an effective inhibitor of the NF-κB pathway [43], was used as a positive control at a concentration of 10 µM and 0.1% DMSO served as vehicle control. The luminescence of the firefly luciferase product and the CTG-derived fluorescence were quantified on a Tecan Spark plate reader (Tecan, Männedorf, Switzerland). The ratio of luminescence units to fluorescence units was calculated to account for differences in cell number. Results were expressed as fold changed relative to the vehicle control with TNFα, which was set to 1 [44]. CTG-fluorescence values used to estimate cell viability were also normalized to the vehicle control with TNFα. Compared to the vehicle control, treatments with fluorescence values below 0.75 were considered as toxic.

#### 2.10.2. Target-Gene Assay

Primary human venous endothelial cells (HUVEC) were isolated from umbilical cords as described previously [8] and maintained in M199 medium (Lonza) supplemented with 20% FCS (Sigma), 2 mM L-glutamine (Sigma), penicillin (100 units/mL), streptomycin (100 mg/mL), 5 units/mL heparin, and 25 mg/mL ECGS (Promocell), and were used up to passage 5. HUVEC were grown to post-confluency in 12-well plates, pre-incubated with PO-E and its microfractions for 30 min, followed by stimulation with 5 ng/mL IL-1β for 90 min. The resorcylic acid lactone of fungal origin (5Z)-7-oxozeaenol [45], TAK1 inhibitor, was used as positive control at a concentration of 5 µM. Total RNA was isolated using the PeqGold Total RNA Isolation Kit (VWR International) according to the manufacturer’s instructions. One µg RNA was reverse transcribed using random hexamers (Fermentas) and murine leukemia virus reverse transcriptase (Thermo Fisher). Real-time PCR was performed with the SsoAdvanced Universal SYBR Green Supermix (BioRad) using the StepOnePlus instrument (Applied Biosystems). The following primer pairs were used (forward/reverse, 5′-3′): E-selectin: CCTGTGAAGCTCCCACTGA/GGCTTTTGGTAGCTTCCATCT; VCAM-1: CCGGCTGGAGATATTAC/TGTATCTCTGGGGGCAA CAT; GAPDH: AGAAGGCTGGGGCTCATTT/CTAAGCAGTTGGTGGTGCAG. Relative mRNA levels were normalized to GAPDH and fold changes calculated according to the 2-ΔΔCT method. Results are shown as mean fold induction of averaged Ct values of triplicates. Cytotoxicity was judged by morphological examination.

### 2.11. Statistical Analysis

NF-κB inhibition data of pure compounds were expressed as the means ± SD of at least three independent biological experiments if not otherwise indicated. All statistical analyses were performed using GraphPad Prism 4.03 software. IC_50_ values were determined by non-linear regression with the sigmoidal dose-response settings (variable slope). Analysis of variance (ANOVA) with Dunnett’s multiple comparison test was used to assess the significant differences between the control and treatment groups. A *p*-value < 0.05 was considered significant. 

## 3. Results

As previously reported, a detannified MeOH extract and a CH_2_Cl_2_ extract prepared from the roots of *P. ostruthium* (both tested at 10 µg/mL) showed an NF-ĸB inhibition of >75% [27]. To unravel the compounds responsible for the pronounced inhibitory activity on this transcription factor, an optimized large-scale extract from the roots and rhizomes of *P. ostruhtium* was prepared. Briefly, the dried plant material was defatted with *n*-hex and the remaining material was subsequently extracted with CH_2_Cl_2_ and MeOH. Both extracts were combined to pool the arsenal of putatively bioactive molecules in one extract labeled as PO-E. The biochemometric approach ELINA [32] was applied to enable a straightforward identification and isolation of the active principles of PO-E. The first objective was to simplify and thus expand the structural complexity of the bioactive extract by the generation of microfractions with quantitative variances of constituents over several consecutive fractions. These were thereupon equally prepared for

(i) ^1^H NMR analysis to obtain quantitative information on structural features of the constituents independent of their ability to ionize (in contrast to an MS-based approach),
(ii) LC-MS-CAD investigation for semi-quantitative information and dereplication of constituents present in each microfraction in both, positive and negative mode, and
(iii) bioactivity testing in three cell-based assays: an NF-ĸB reporter-gene assay and two functional assays quantifying mRNA expression of NF-ĸB target-genes (E-selectin and VCAM-1).

**Figure 1:**
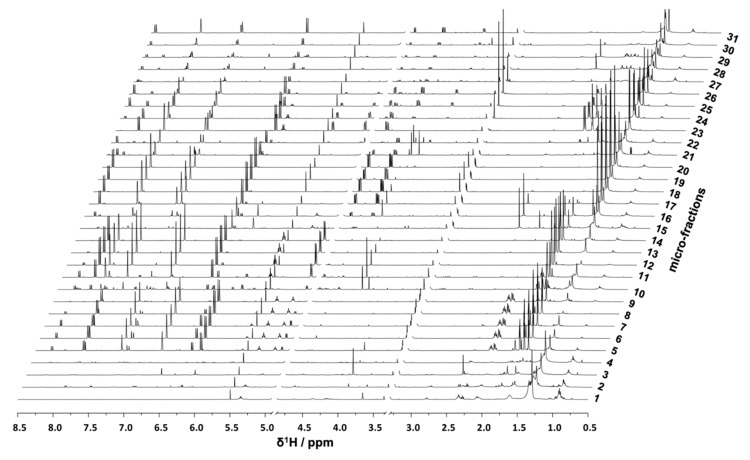
Stack Plot of PO01_01–PO01_31; ^1^H NMR data (δ_H_ 0.5–8.5) of obtained *Peucedanum ostruthium* microfractions after fractionation with high-performance counter-current chromatographic (HPCCC). For clarity reasons, both, the water signal (at 4.9 ppm) and the signal of the solvent (at 3.31 ppm) are not shown.

**Figure 2:**
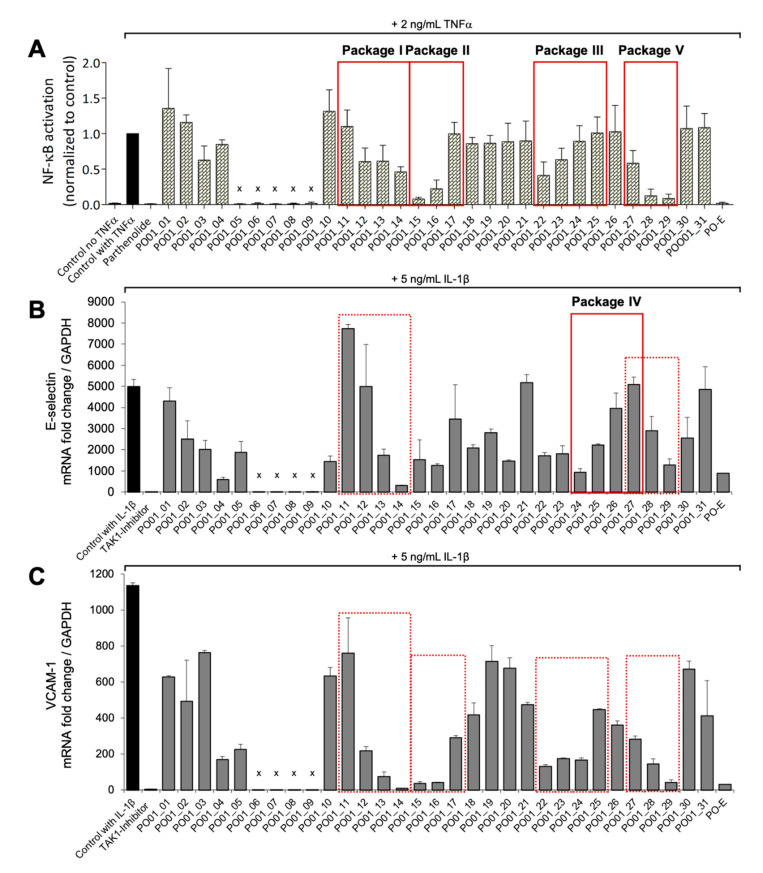
Bioactivity data of PO01_01–PO01_31 in (A) an NF-ĸB reporter-gene assay, a real-time PCR analysis of (B) E-selectin and (C) VCAM-1. Packages I–V are shown as red squares (solid lines, bioactivity data used for HetCA analysis; dotted lines, packages for comparison of bioactivity data); (**A**) PO01_01–PO01_31, the crude extract PO-E (all 10 µg/mL) and the positive control parthenolide (10 µM) were tested in a cell-based in vitro NF-κB-driven luciferase reporter assay in HEK293–NF-κB-luc cells stimulated with 2 ng/mL TNFα as indicated. The luciferase signal derived from the NF-κB reporter was normalized to the CTG-fluorescence and expressed as fold change normalized to the vehicle control (DMSO 0.1%) with TNFα. Bar charts represent (residual) NF-κB transactivation activity expressed as mean ± SD, *n* = 3. Microfractions marked with an “x” showed cytotoxicity; (**B**) PO01_01–PO01_31, the crude extract PO-E (all 50 µg/mL), the positive control TAK1-inhibitor (5 µM (5Z)-7-oxozeaenol) and vehicle control (DMSO 0.5%) were assayed for E-selectin expression in primary human venous endothelial cells (HUVEC). Real-time PCR of cDNA obtained from IL-1β (5 ng/mL, 90 min)-stimulated HUVEC which were either untreated (DMSO) or pre-treated (30 min) with 50 µg/mL PO01_01-PO01_31. Relative mRNA levels of E-selectin were normalized to GAPDH and expression levels are depicted as mean fold change ± SD compared to non-stimulated cells. *n* = 3. Microfractions marked with an “x” showed cytotoxicity; (**C**) PO01_01-PO01_31, PO-E (all 50 µg/mL), the positive control TAK1-inhibitor (5 µM) and vehicle control (DMSO 0.5%) were assayed for VCAM-1 expression in primary HUVEC. Real-time PCR of cDNA obtained from IL-1β (5 ng/mL, 90 min)-stimulated HUVEC, which were either untreated (DMSO) or pre-treated (30 min) with 50 µg/mL PO01_01–PO01_31. Relative mRNA levels of VCAM-1 were normalized to GAPDH and expression levels are depicted as mean fold change ± SD compared to non-stimulated cells, *n* = 3. Microfractions marked with an “x” showed cytotoxicity.

For microfractionation, an HPCCC with gradient elution in normal-phase mode was employed. An HPCCC method was developed allowing a high-resolution efficiency able to fractionate the crude extract in one single fractionation step. PO-E was fractionated by applying seven different two-phase solvent systems composed of *n*-hex/EtOAc/MeOH/H_2_O with increasing polarity without stopping the apparatus (Table S1). By applying this optimized technique, an efficient fractionation of *P. ostruthium* constituents was guaranteed in a single operation by HPCCC. In total, more than 565 tubes were collected and pooled to 31 microfractions (PO01_01–PO01_31) according to their TLC fingerprint. Aliquots of PO01_01-PO01_31 were forwarded to ^1^H NMR analysis, LC-MS-CAD measurements and bioactivity testing in an NF-ĸB reporter-gene assay using HEK293 cells, and two functional target-gene assays (E-selectin and VCAM-1) using endothelial cells. In parallel to a quantitative variance of ^1^H NMR signals over consecutive microfractions (Figure 1), bioactivity patterns of three cell-based assays relating to this variation were obtained for PO01_01 to PO01_31 (Figure 2). 

**Figure 3:**
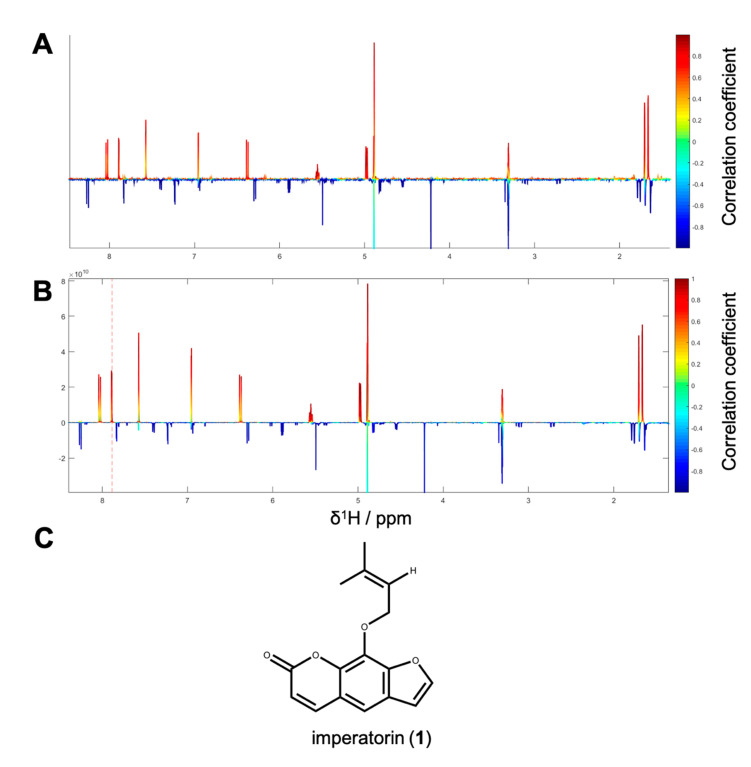
(**A**) HetCA plot of package I. The color code is based on the correlation coefficient: red signals (“hot features”) are positive, blue signals (“cold features”) are negatively correlated with bioactivity; (**B**) STOCSY plot of package I. The signal at δ_H_ 7.88 was chosen to obtain the information which molecule(s) share this hot feature. The plot is color coded based on the correlation coefficient: red = signals belonging to molecule(s) that have a signal at δ_H_ 7.88; blue = signals belong to molecule(s) that do not have the signal at δ_H_ 7.88; (**C**) structure of the positively correlated and identified compound **1.**

By taking a closer look at the bioactivity results, it became obvious that the activity profile of the microfractions in the NF-ĸB reporter gene assay shows a very similar pattern as the activity profiles in the two NF-κB target-gene assays. For instance, PO01_06 to PO01_09 showed cytotoxicity in all assays. Further, an increase in activity was observed from PO01_11 to PO01_14, whereas a decrease was obvious from PO01_27 to PO01_29. Increasing activity was shown for PO01_01 to PO01_03 in the NF-ĸB reporter-gene assay and the NF-ĸB target-gene assay (E-selectin). Likewise, a similar bioactivity pattern was observed for PO01_15 to PO01_17 in the NF-ĸB reporter-gene assay and the NF-ĸB target-gene assay (VCAM-1). Here, a decreasing activity can be observed throughout these fractions. Interestingly, by perceiving the bioactivity results for PO01_22 to PO01_25, a decreasing activity could be shown for the reporter-gene assay and the target-gene assay (VCAM-1). In this package, the active principle seems to be comprised of PO01_22 rather than in PO01_24. On the contrary, in the target-gene assay quantifying E-selectin mRNA expression an explicit decrease in activity from PO01_24 to PO01_27 is shown, indicating that the active constituent(s) is/are accumulated in PO01_24 rather than in PO01_27. This decrease in activity for PO01_24 to PO01_26 was also shown in the target-gene assay when tested at 50 µg/mL (Figure S2). Because of the high correlation between the inhibition of NF-ĸB transactivation activity and the expression of both adhesion molecules on the mRNA level, the biochemometric correlations were predominantly elaborated based on the bioactivity data of the NF-ĸB reporter-gene assay. For ELINA, the concentration variances of compounds comprised in the microfractions of a chosen package (e.g., also reflected in their ^1^H NMR data) were correlated with the bioactivity data by using the multivariate statistical tool HetCA as described before [32]: HetCA plots were generated to visualize the correlation between ^1^H NMR spectra with their corresponding bioactivity data. Therefore, packages of three to four consecutive microfractions with a variance in activity were selected and depicted as HetCA plots. By applying this method, structural features of molecule(s) correlating to bioactivity could already be seen at the early stage of phytochemical workup, i.e., after the single fractionation step of PO-E. Hence, the HetCA plot of package I (i.e., PO01_11–PO01_14) (Figure 3A) displays structural features of molecule(s) correlating with NF-ĸB inhibition. Features belonging to ^1^H NMR signals exhibiting a positive correlation with NF-ĸB inhibition (red) were assigned as “hot” features whereas, features belonging to ^1^H NMR signals with a negative correlation with NF-ĸB inhibition (blue) were assigned as “cold” features. Thus, it became evident that aromatic compounds giving resonances in the downfield chemical shift area (such as coumarins or furanocoumarins) contribute to the inhibition of the transcription factor NF-ĸB. Further, statistical total correlation spectroscopy (STOCSY) analysis was implemented to deliver information in which molecule(s) share specific “hot” features [42]. For instance, the HetCA plot of package I (Figure 3A) resembles the STOCSY plot of package I (Figure 3B) indicating that there is only one molecule responsible for the observed anti-inflammatory activity. By taking a closer look on the generated STOCSY plot, it can be seen that the molecule with “hot” features gives five aromatic proton signals (between δ_H_ 6.00–9.00) with an aliphatic side chain: at δ_H_ 1.67 and 1.71 two singlets can be seen typical for methyl groups, a triplet at δ_H_ 5.55 typically given by a vinylic proton as well as a doublet at δ_H_ 4.97. Further, the signal of water and the solvent are present at 4.87 and 3.31 ppm. LC-MS was used to facilitate the identification of the bioactive molecule via dereplication. By the additional use of a charged aerosol detector (CAD), a semi-quantitative analysis could be performed and allowed for the visualization of increasing or decreasing peak areas under the curve (AUC) within a package. By implementing this information, further correlation with bioactivity could be achieved. For instance, an overlay of the four chromatograms of package I revealed a continuous increase of the AUC of the peak at the LC retention time (t_R_) 7.6 min from the least active microfraction PO01_11 to the most active PO01_14 (Figure S3). A dereplication of the selected peak with a *m/z* value of 271.13 g/mol in the positive mode identified the furanocoumarin imperatorin (**1**) [28] as the bioactive constituent without any preceding isolation efforts. Figure 3C shows that the structural predictions delivered by the STOCSY plot match with the actual structure of the predicted molecule. Here, no targeted isolation was performed as **1** was already in-house available as a pure compound. LC-MS-CAD analysis was performed to compare the LC chromatogram and MS spectra of the microfraction PO01_14 and compound **1** (Figure S4A,B).

**Figure 4:**
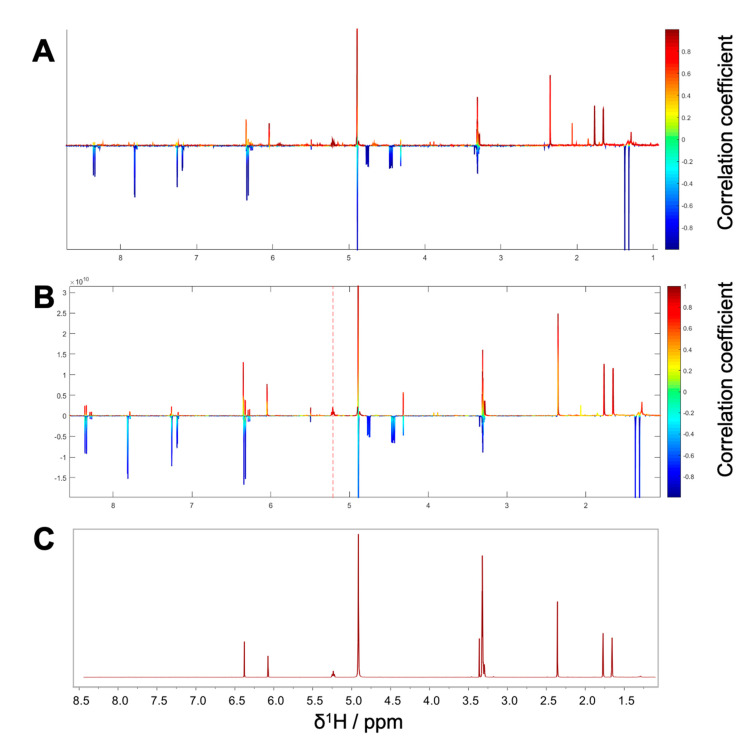
Package II (**A**) ^1^H NMR pseudo-spectrum showing the HetCA of ^1^H NMR spectra and NF-ĸB reporter-gene inhibition data of the microfractions PO01_15–PO01_17. (**B**) STOCSY plot of package II. The signal at δ_H_ 5.21 was chosen to obtain the information in which molecule(s) share this hot feature. (**C**) ^1^H NMR spectra of the isolated active compound **2**.

Package II was composed of the microfractions PO01_15 to PO01_17, since an explicit decline in activity was observed in both, the NF-ĸB reporter-gene assay and the target-gene assay on VCAM1. The HetCA pseudo spectrum and the STOCSY plot of package II revealed that at least two molecules contribute to the observed activity (compare Figure 4A,B): the STOCSY plot at δ_H_ 5.21 displays a molecule with two aromatic signals (singlets at δ_H_ 6.35 and 6.05), whereas the signal at δ_H_ 5.21 is given by a vinylic proton. Two methyl protons are further shown at δ_H_ 1.66 and 1.77 as well as a doublet at δ_H_ 3.29. The prominent signal at δ_H_ 2.35 indicates either the presence of a benzylic proton or a carbonyl methyl group. Apart from this, further signals in the downfield chemical shift area are present in the HetCA pseudo spectrum given by four doublets at δ_H_ 8.03, 7.89, 6.96, and 6.38 as well as a singlet at δ_H_ 7.57 (compare with Figure 3B). Besides the NMR-bioactivity correlation, a dereplication via LC-MS-CAD of package II was performed. In the most active fraction, PO01_15, two peaks at t_R_ 7.3 min and 7.6 min (red squares) are shown (Figure S5). The peak at t_R_ 7.6 min is the previously identified compound **1**, responsible for the activity of package I. The second peak in the most active microfraction PO01_15 present at t_R_ 7.30 min contains a *m/z* value of 261.28 in the positive mode. A dereplication via a literature search was performed under consideration of (i) the molecular weight of the compound at t_R_ 7.30 min and (ii) the structural information derived from the STOCSY plot. By this, the chromone peucenin (**2**) was identified as an active principle in package II. A targeted isolation and structure elucidation of **2** confirmed the ELINA prediction of package II (compare Figure 4B,C). Likewise, the furanocoumarin oxypeucedanin (**3**) [28] was identified as an inactive principle of package II (Figure S5; blue square).

**Figure 5:**
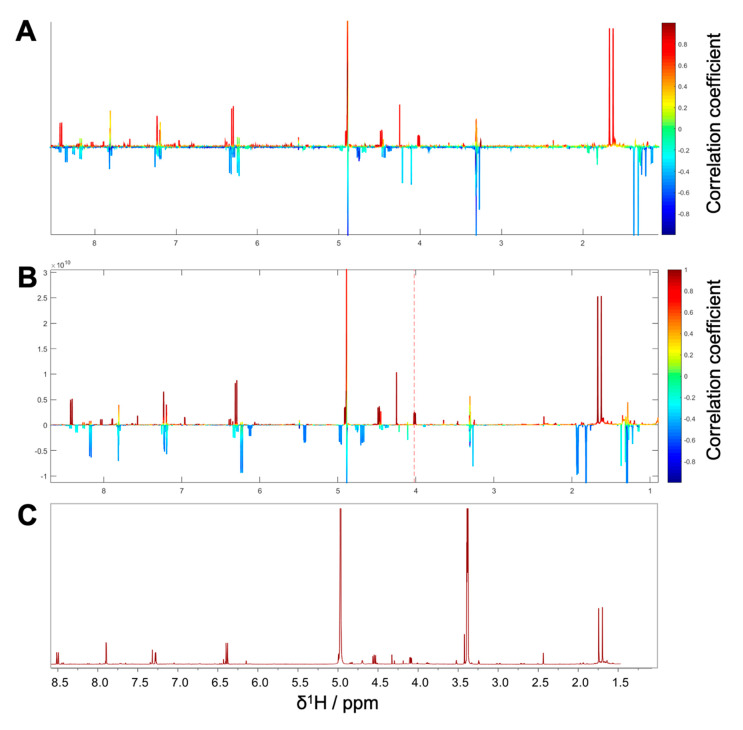
Package III (**A**) HetCA plot of PO01_22–PO01_25. (**B**) STOCSY plot; the signal at δ_H_ 4.03 was chosen to obtain the information which molecule(s) share this hot feature and (**C**) ^1^H NMR spectra of the isolated active compound **4**.

The microfractions PO01_22 to PO01_25 were selected for the generation of package III. Here, the STOCSY plot (δ_H_ 4.0; Figure 5B) strongly resembles the HetCA plot of package III (Figure 5A), indicating that there is mainly one molecule responsible for the observed activity in the reporter-gene assay and target-gene assay (VCAM-1). The bioactive molecule shows resonances in the downfield chemical shift area, typical for the aromatic backbone of furanocoumarins (four doublets at δ_H_ 8.42, 7.81, 7.20, and 6.31 as well as a singlet at δ_H_ 7.24), two resonances in the upfield resonance area given by aliphatic protons such as methyl groups (δ_H_ 1.67 and 1.63). Further signals are present at δ_H_ 4.62 (singlet), 4.47 and 4.02 (doublet of doublet). A semi-quantitative LC-MS-CAD analysis allowed for the visualization of the concentration differences of the respective peaks within package III (Figure S6). The chromatogram revealed an increase of the AUC for the peak at t_R_ 7.33 min from the most active microfraction PO01_22 to PO01_24 (thereupon decreasing from PO01_24 to PO01_25). The peak at t_R_ 6.01 min is not present in PO01_22, whereas the peak at t_R_ 6.98 min is only present in the most active microfraction PO01_22. A dereplication for the respective peak revealed a *m/z* value of 323.12 in the positive mode. Further, an MS^2^ fragmentation pattern of –35 g/mol was observed for this selected peak, typical for the halogene chlorine. Under consideration of the molecular weight, the fragmentation pattern and the hot features from the STOCSY plot, literature research was performed and unveiled the furanocoumarin saxalin, however with undefined stereochemistry (**4**) [46] as bioactive compound. Targeted isolation and structure elucidation with 1D and 2D NMR experiments (expect for NOESY) were performed and confirmed the presence of the chlorinated compound **4** within the most active microfraction PO01_22. 

**Figure 6:**
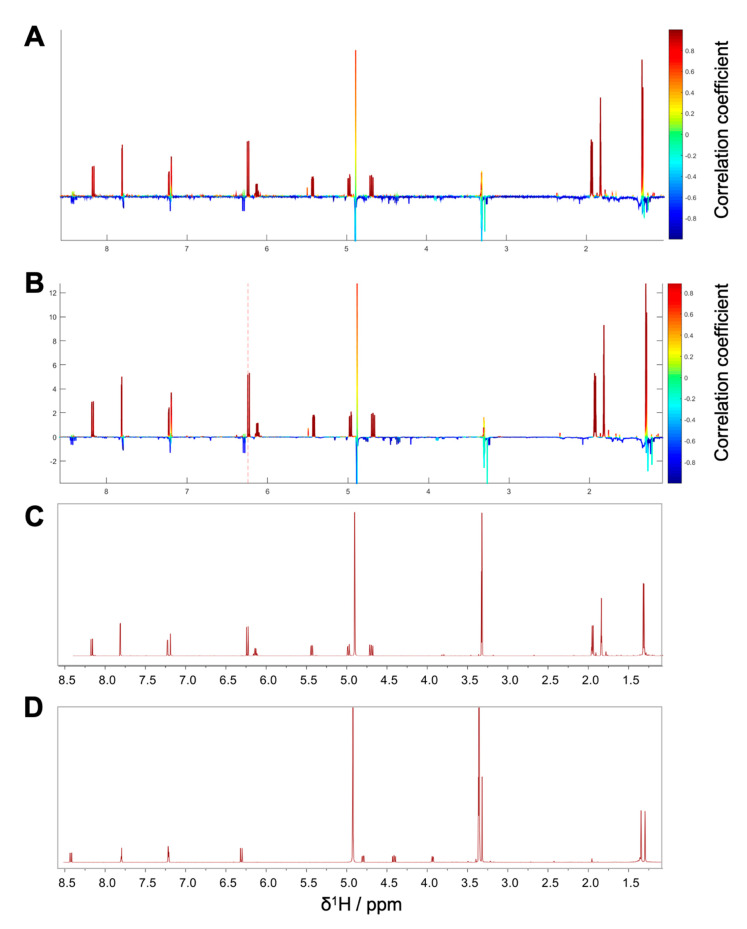
(**A**) ^1^H NMR pseudo-spectrum showing the HetCA of ^1^H NMR spectra and NF-ĸB target-gene assay on E-selectin of selected fractions PO01_24–PO01_27. (**B**) STOCSY plot of package IV. The signal at δ_H_ 6.24 was chosen to obtain the information in which molecule(s) share this hot feature. (**C**) ^1^H NMR spectra of the isolated active compound **5** and (**D**) ^1^H NMR spectra of the isolated compound **6** predicted as inactive.

As the microfractions PO01_24 to PO01_27 exhibited a decreasing activity (i) on the expression of E-selectin in the target-gene assay, (ii) in the reporter-gene assay at 50 µg/mL (Figure S2) and (iii) PO01_24 showed a similar activity than the preceding microfractions PO01_22 and PO01_23 on the expression of VCAM-1, these microfractions were used for the generation of package IV. Here, the ELINA approach unveiled the furanocoumarin ostruthol (**5**) [28] as the active principle (Figure 6A,B; red signals). Targeted isolation of **5** was performed; additionally, a second compound (**6**) was co-isolated showing cold features. Following the structure elucidation confirmed the isolated compound as **5** by using 1D and 2D NMR experiments. Compound **6** was identified as oxypeucedanin methanolate [47] which was negatively correlated with activity (Figure 6C,D).

**Figure 7:**
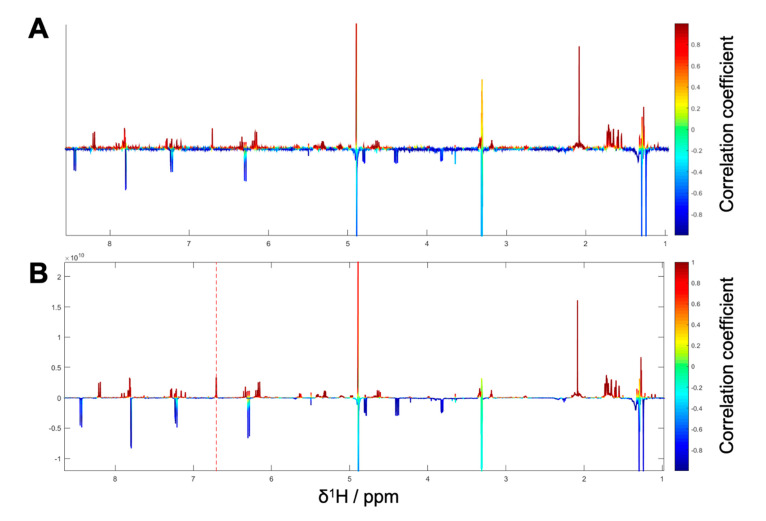
(**A**) ^1^H NMR pseudo-spectrum showing the HetCA of ^1^H NMR spectra and NF-ĸB reporter-gene assay of selected fractions PO01_27–PO01_29 (i.e., package V); (**B**) STOCSY plot of package V. The signal at δ_H_ 6.71 was chosen to obtain the information which molecule(s) share this hot feature.

**Figure 8:**
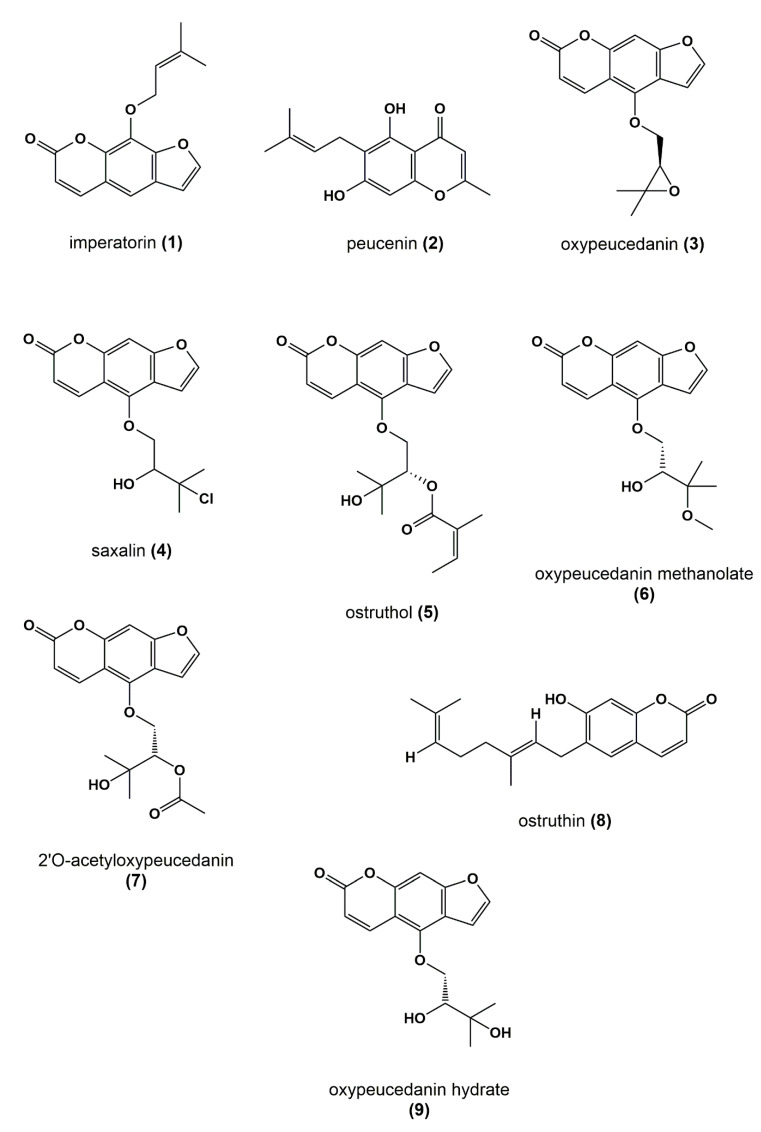
Chemical structures of identified *P. ostruthium* constituents.

The last package analyzed was generated from PO01_27 to PO01_29, i.e., package V. Here, ELINA unveiled the presence of two compounds responsible for the observed bioactivities in all three assays, i.e., the furanocoumarin 2’-O-acetyloxypeucedanin (**7**) [46] and the coumarin ostruthin (**8**). Further, the furanocoumarin oxypeucedanin hydrate (**9**) [28] was predicted as an inactive compound (Figure 7A,B; cold features). Because of the low quantity of the microfraction PO01_29 no isolation was performed of **7** and **8**, whereas compound **9** was available as an in-house pure compound (Figure 8).

**Figure 9:**
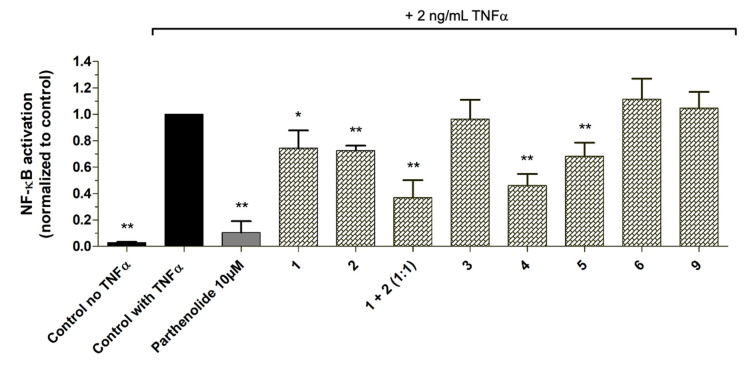
Residual NF-ĸB activity (normalized to the vehicle control with TNFα) in TNFα (2 ng/mL)-activated HEK293–NF-ĸB-luc cells after treatment with the compounds **1**–**6**, **9** and a 1:1 mixture of **1** and **2** (all tested at 10 µg/mL) and the positive control parthenolide (10 µM). The luciferase signal derived from the NF-ĸB reporter was normalized to CTG-fluorescence and expressed as fold change normalized to the vehicle control (0.1% DMSO) with TNFα. Bar charts represent (residual) NF-ĸB transactivation activity expressed as mean ± SD. *n* = 4; ∗ *p* < 0.05 and ∗∗ *p* < 0.01 as compared to the control with TNFα.

As proof of concept, the isolated pure compounds **2**, **4**, **5,** and **6**, as well as the in-house available compounds **1, 3**, and **9** were tested in the NF-κB reporter gene assay. Additionally, **1** and **2** were also tested as a mixture (1:1) as they were both predicted to contribute to the activity in the most active microfraction PO01_15 (Figure 9). Further, dose-response experiments were performed with positively correlated compounds and isolates **1**, **2**, **4,** and **5**, respectively (Figure S7). Compounds **1** and **2**, when tested separately, showed only weak NF-ĸB inhibitory activities with an IC_50_ value of 49.0 µM for **1.** For compound **2** dose-response experiments revealed hardly any concentration-dependent activity resulting in a rather flat curve. However, when tested as a mixture, a significant increase of the inhibitory effect was shown (*p* < 0.01) in the NF-ĸB reporter-gene assay (Figure 9**)**. Whereas **5** exerted a moderate inhibitory activity (with an IC_50_ value of about 20 µM), **4** was identified as potent NF-κB inhibiting compound (*p* < 0.01) with an IC_50_ value of 8.08 µM. In line with the ELINA prediction, the negatively correlated compounds **3**, **6,** and **9**, showed no bioactivity, when tested in the NF-κB reporter-gene assay at 10 µg/mL, and thus confirmed the accuracy of the presented biochemometric approach. 

## 4. Discussion

In this study, we applied a biochemometric approach to unravel those constituents, which contribute to the NF-ĸB inhibitory activity in the masterwort extract PO-E using the recently established ELINA approach [37]. As a first and crucial step, a newly elaborated protocol for a comprehensive gradient-elution HPCCC was developed for the microfractionation of PO-E. This enabled an appropriate deconvolution of the crude extract without compound(s) adhesion to any chromatographic material, and accordingly without the risk of putatively losing bioactives. In this way, the complexity of PO-E was broken down to 31 microfractions with envisaged concentration variations of constituents. 

Bioactivity data of three in vitro cell-based assays addressing the NF-ĸB inhibitory activity in masterwort were acquired for all 31 microfractions. Here, the implementation of in vitro cell-based assays in the biochemometric ELINA approach has substantial benefits. First, in contrast to cell-free assays, they offer the advantage to model the biology of intact cells. Thus, not only the activity data of a sample can be shown, but also biologically relevant information like the effect of the sample on cell viability and cytotoxicity can be elucidated. Second, molecular pathway interactions can be exposed [34,48,49]. Although some biochemometric studies have previously been performed with e.g. enzyme-based assays to decipher bioactives in complex mixtures [32,36,37,38], the present study aimed to evaluate the robustness of the ELINA approach applying a cell-based readout to correlate with big metabolite data derived from NMR and MS. Indeed, all the positively correlated constituents tested in the NF-ĸB reporter-gene assay (i.e., **1**, **2**, **4**, **5**) showed significant inhibitory activities (Figure 9), whereas the negatively correlated compounds (i.e., **3**, **6,** and **9**) revealed as inactive (tested at 10 µM). In addition, all the above tested compounds were well tolerated in the assay and showed no in vitro cytotoxicity (Figure S8). With the scope of this study, the application of a biochemometric approach in cell-based in vitro assays on the transcription factor NF-ĸB was successfully demonstrated.

Pharmacological effects given by a crude extract are often a result of the combination of constituents rather than individual chemical entities out of that mixture. Traditional medicinal systems like the European phytotherapy, traditional Chinese medicine, and Ayurveda, however, rely on multi-component mixtures instead of single compounds to treat pleiotropic diseases [50] such as inflammation. Identifying multiple compounds that contribute additively, synergistically or antagonistically to a biological effect remains a challenging task in NP drug discovery [34,51]. To unravel bioactive compound(s) from a complex mixture, metabolomics approaches have been introduced in the past few years. They offer a more holistic perspective on bioactives [36,52] by profiling multiple mixture components simultaneously [30]. Using the example of masterwort, additive effects of compounds **1** and **2** were observed within the most active microfraction PO01_15. This effect was imitated when **1** and **2** were tested as 1:1 mixture (Figure 9). On the contrary, when taking a closer look at the LC-MS chromatograms of package I (i.e., PO01_11-PO01_14; Figure S3), the detrimental effects of several compounds (present at t_R_ 7.8, 8.3, and 9.3 min) within PO01_11 are assumed. Several compounds seem to antagonize the activity of **1** (present at t_R_ 7.6 min in PO01_11-PO01_14). This conclusion is in line with the quantitative ^1^H NMR data of package I (Figure 1; for more details see Figure S9): in all microfractions resonances given by **1** are present. These findings emphasize a unique strength of the ELINA approach: combinatorial effects of constituents that would probably have been missed using the classical bioactivity-guided isolation approach are disclosed from mixtures. Moreover, these positively correlated compounds can be identified prior to any isolation, thus avoiding unnecessary and tedious isolation procedures. 

## 5. Conclusion

The biochemometric approach ELINA enabled a targeted identification of the NF-ĸB inhibiting metabolites of the traditionally used masterwort extract after one single fractionation step. Concentration variances of HPCCC microfractions unmasked the anti-inflammatory constituents probed in an NF-κB reporter-gene assay and two NF-κB target-gene assays quantifying VCAM-1 and E-selectin mRNA. In sum, three furanocoumarins (**1**, **4**, and **7**), one chromone (**2**) and one coumarin (**8**) were pinpointed as NF-ĸB inhibitory agents present in *P. ostruthium* even prior to any isolation. As proof of concept, the positively correlated compounds **1**, **2**, **4** and **5** as well as the negatively correlated compounds **3**, **6,** and **9** were tested in the cell-based NF-ĸB reporter-gene assay and confirmed the prediction. Intriguingly, ELINA enabled to unravel additive effects of compounds **1** and **2** that would have probably been missed in a classic bioactivity-guided isolation procedure. By applying this biochemometric approach, early identification and dereplication of even minor bioactives were achieved. Thus, this holistic –omics-based tool not only offers insight into the chemical features and those metabolites contributing to the bioactivity of multicomponent NPs but also has the potential to effectively accelerate the NP based drug discovery process.

## Supplementary Materials

The following are available online at https://www.mdpi.com/2218-273X/10/5/679/s1. **Table S1:** HEMWat system table for HPCCC fractionation with gradient elution. **Table S2:** Pooling of microfractions. **Figure S1:** TLC of microfractions PO01_01-PO01_31; detection UV_254_ (top) and UV_366_ (bottom). **Figure S2** Bioactivity results of PO01_11-PO01_29, the crude extract PO-E (all 50 µg/mL) and the positive control parthenolide (10 µM) tested in a cell-based in vitro NF-ĸB-driven luciferase reporter assay in HEK293–NFKB-luc cells stimulated with 2 ng/mL TNFα as indicated. The luciferase signal derived from the NF-ĸB reporter was normalized to the vehicle control (0.1% DMSO) with TNFα. Bar charts represent (residual) NF-ĸB transactivation activity expressed as mean ± SD; *n* = 3. **Figure S3:** CAD chromatogram of package I (PO01_11–PO01_14); t_R_ 6.00–9.60 min. **Figure S4:** LC-MS-CAD analysis of the microfraction PO01_14 and the pure compound imperatorin; (**A**) LC chromatogram of PO01_14 and imperatorin at t_R_ 7.6 min; (**B**) Mass spectra of PO01_14 and imperatorin showing the *m/z* value of 271.1 g/mol. **Figure S5**: CAD chromatogram of package II (PO01_14-PO01_17); t_R_ 6.0–9.0 min. **Figure S6:** CAD chromatogram of package III (PO01_22-PO01_25); t_R_ 5.0–8.5 min. **Figure S7:** Concentration-response curves showing the positively correlated compounds 1, 2, 4 and 5 assayed in a cell-based NF-κB-reporter gene assay at different concentrations. Shown are the residual NF-ĸB activities in TNFα (2 ng/mL)-activated HEK293–NF-ĸB-luc cells after treatment with the respective compounds. The luciferase signal derived from the NF-ĸB reporter was normalized to the CTG-fluorescence and expressed as fold change normalized to the TNFα signal. IC_50_ values were determined by non-linear regression with the sigmoidal dose response settings (variable slope) using GraphPad Prism 4.03 software. **Figure S8**: CTG fluorescence as a measure for cell viability after treatment with pure compounds 1–6, 9 and a 1:1 mixture of 1 and 2 (all tested at 10 µg/mL) and the positive control parthenolide (10 µM) in TNFα (2 ng/mL)-activated HEK293–NF-ĸB-luc cells; data are normalized to vehicle control (0.1% DMSO) with TNFα. Data represent mean ± SD. *n* = 4. **Figure S9:** ^1^H NMR data of package I (PO01_11–PO01_14).
