# Supplementary material for: A Biochemometric Approach for the Identification of In Vitro Anti-Inflammatory Constituents in Masterwort
Source: Biomolecules. 2020 Apr 28;10(5):679. doi: 10.3390/biom10050679 (PMC7277629; doi:10.3390/biom10050679)
Supplement: Supplementary file 1 [file biomolecules-10-00679-s001.pdf]

biomolecules

Supplementary Information

A Biochemometric Approach for the Identification of In Vitro Anti-Inflammatory Constituents of Masterwort

Julia Zwirchmayr [1], Ulrike Grienke [1], Scarlet Hummelbrunner [1], Jaqueline Seigner [2], Rainer de Martin [2], Verena M. Dirsch [1], and Judith M. Rollinger [1,*]

[1] Department of Pharmacognosy, Faculty of Life Sciences, University of Vienna, Althanstraße 14, 1090 Vienna, Austria
[2] Department of Vascular Biology and Thrombosis Research, Medical University of Vienna, Schwarzspanierstaße. 17, 1090 Vienna, Austria
* Correspondence: judith.rollinger@univie.ac.at; Tel.: +43-1-4277-55255; Fax: +43-1-4277-855255

Received: date; Accepted: date; Published: date

Table S1

Table S1 HEMWat system table for HPCCC fractionation with gradient elution

HEMWat System No.	Solvent ratios				Used layer of binary solvent system
	n-hex	EtOAc	MeOH	H_2O	
22	3	1	3	1	upper & lower layer
21	5	2	5	2	upper layer
20	2	1	2	1	upper layer
19	3	2	3	2	upper layer
17	1	1	1	1	upper layer
15	2	3	2	3	upper layer
10	1	5	1	5	upper & lower layer

Table S2

Table S2 Pooling of microfractions

HPCCC Fractions of 1st and 2nd run		Fraction Yield [mg]	Microfraction
1st run	2nd run		
1 – 13	1 – 11	64.09	PO01_01
14 – 20	12 – 17	10.13	PO01_02
21 – 25	18 – 23	5.23	PO01_03
26 – 36	24 – 26	4.21	PO01_04
37 – 41	27 – 28	9.86	PO01_05
42 – 44	29 – 31	58.44	PO01_06

45 – 46	32 – 36	38.64	PO01_07

**HPCCC Fractions of 1st and 2nd run**		**Fraction Yield [mg]**	**Mircofraction**
1st run	2nd run		
47 – 49	-	20.75	PO01_08
50 – 52	-	9.01	PO01_09
53 – 55	37 – 55		
56 – 66		11.34	PO01_10
67 – 72			
73 – 83	56 – 57	8.34	PO01_11
84 – 91	-	9.30	PO01_12
92 – 99	58 – 60	13.32	PO01_13
100 – 105	61 – 68	12.59	PO01_14
106 – 109	69 – 86	7.61	PO01_15
110 – 114			
115 – 119		7.77	
120 – 125	87 – 95		PO01_16
126 – 131	96 – 100	7.57	PO01_17
132 – 134	101 – 104	6.05	PO01_18
135 – 138	105 – 109	9.16	PO01_19
139 – 142	110 – 114	6.14	PO01_20
143 – 146	115 – 122	5.21	PO01_21
147 – 150			
151 – 155	123 – 133		
156 – 166	134 – 144	7.21	PO01_22
167 – 172	145 – 155		
176 – 178	169 – 179	10.69	PO01_23
173 – 175	156 – 168	13.39	PO01_24
179 – 181	180 – 191	16.11	PO01_25
182 – 184	192 – 200	8.35	PO01_26
185 – 188	201 – 212	4.80	PO01_27
189 – 192	213 – 216	5.56	PO01_28
193 – 197	217 – 222	11.37	PO01_29
198 – 202	223 – 225	4.77	PO01_30
203 – 206	226 – 280	40.04	PO01_31
207 – 212	-		
213 – 217	-		
218 – 223	-		
224 – 228	-		
229 – 232	-		
233 – 238	-		
239 – 285	-		

Figure S1

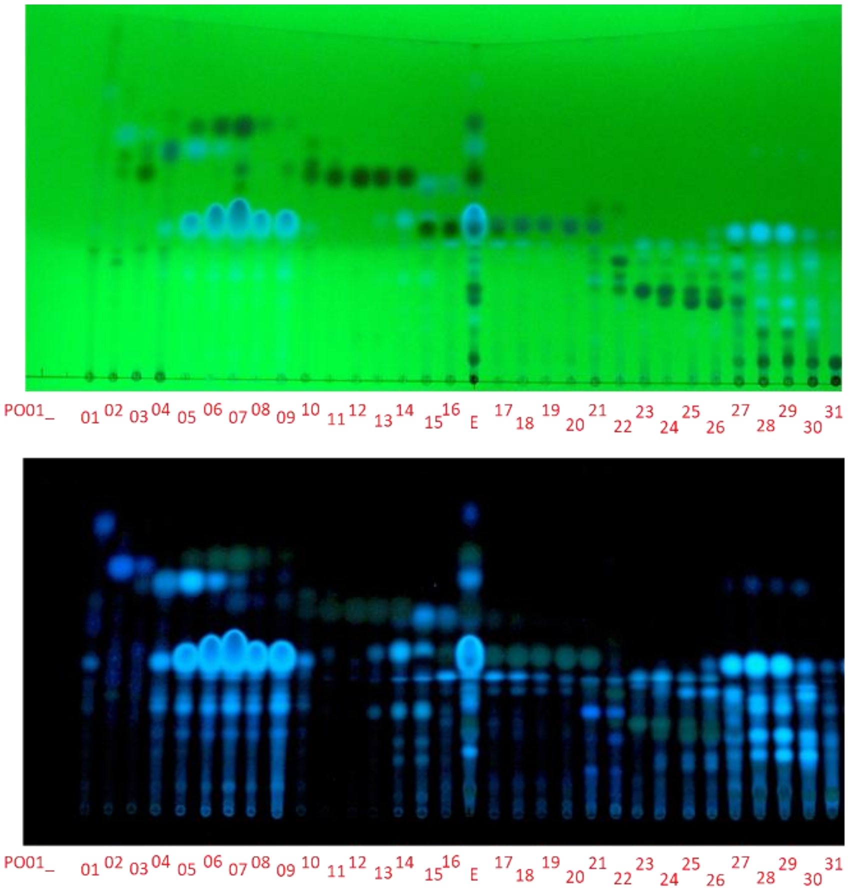

Figure S1 TLC of microfractions PO01_01-PO01_31; detection UV254 (top) and UV366 (bottom).

Figure S2

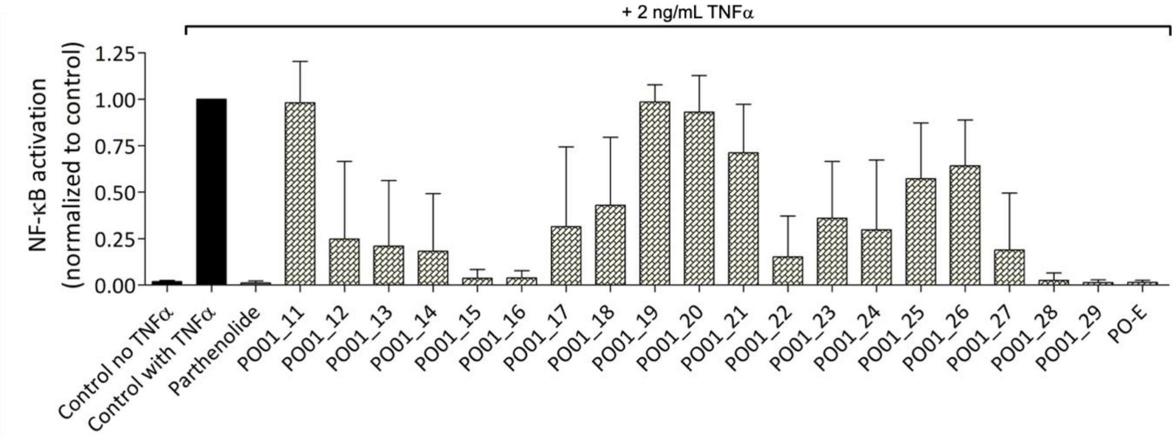

Figure S2. Bioactivity results of PO01_11-PO01_29, the crude extract PO-E (all 50 µg/mL) and the positive control parthenolide (10 µM) tested in a cell-based in vitro NF-κB-driven luciferase reporter assay in HEK293-NFκB-luc cells stimulated with 2 ng/mL TNFα as indicated. The luciferase signal derived from the NF-κB reporter was normalized to the vehicle control (0.1% DMSO) with TNFα. Bar charts represent (residual) NF-κB transactivation activity expressed as mean ± SD; *n* = 3.

Figure S3

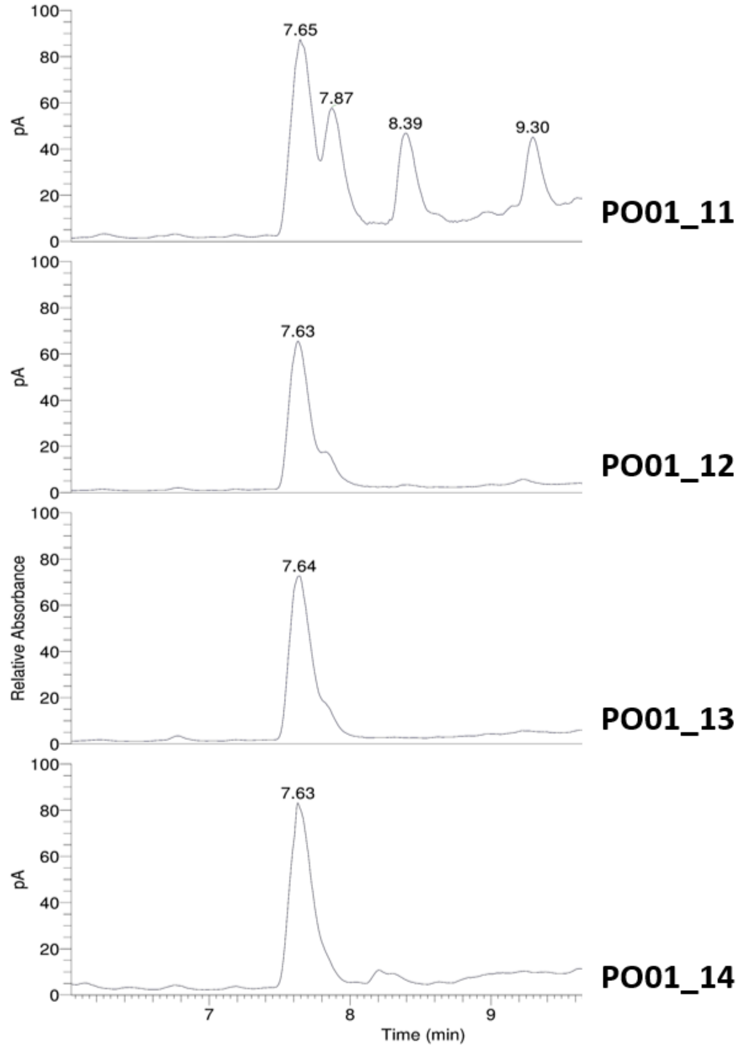

Figure S3. CAD chromatogram of package I (PO01_11–PO01_14); tR 6.00 – 9.60 min.

Figure S4

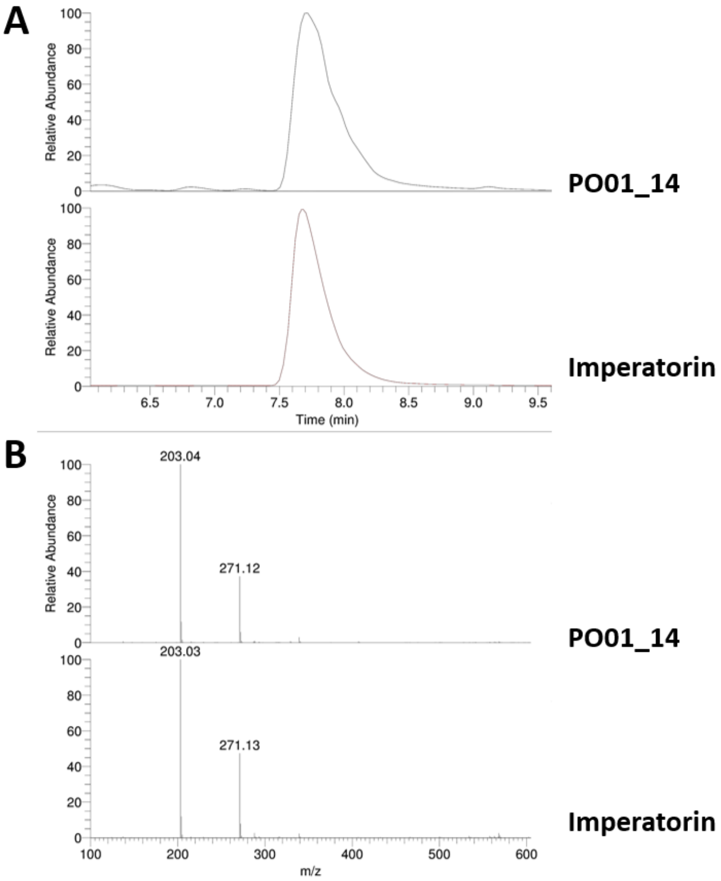

Figure S4. LC-MS-CAD analysis of the microfraction PO01_14 and the pure compound imperatorin; (**A**) LC chromatogram of PO01_14 and imperatorin at t_R 7.6 min; (**B**) Mass spectra of PO01_14 and imperatorin showing the *m*/*z* value of 271.1 g/mol.

Figure S5

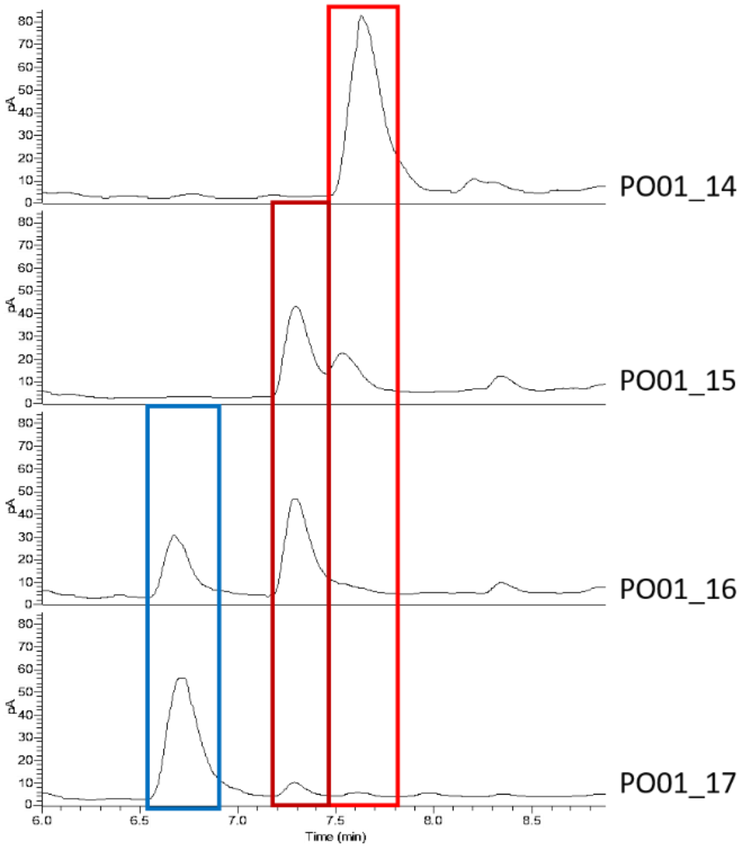

Figure S5. CAD chromatogram of package II (PO01_14-PO01_17); t_R 6.0–9.0 min.

Figure S6

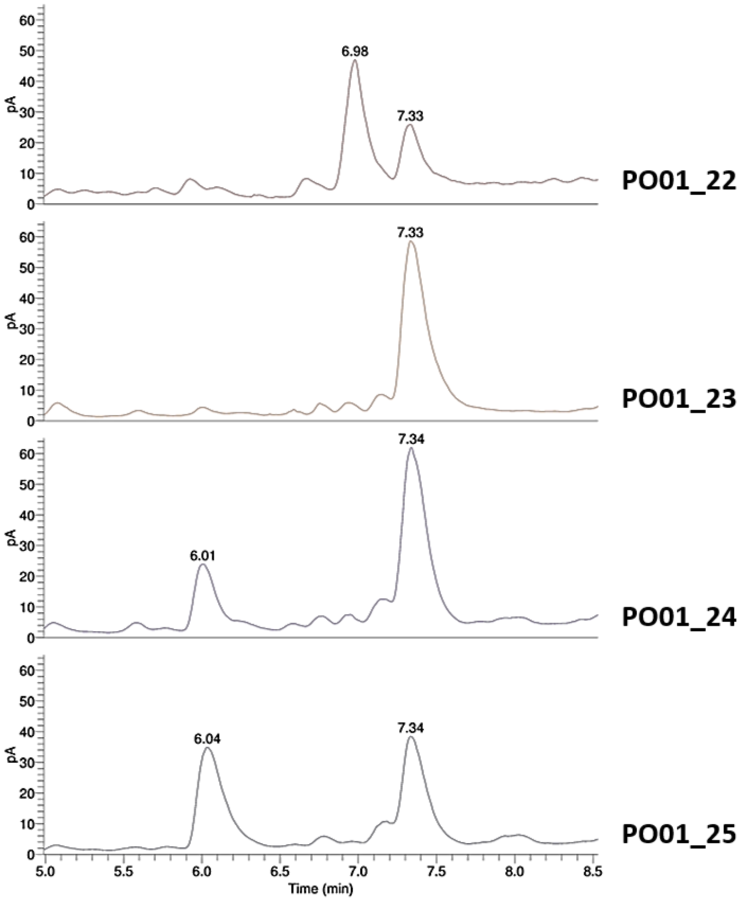

Figure S6. CAD chromatogram of package III (PO01_22-PO01_25); t_R 5.0–8.5 min.

Figure S7

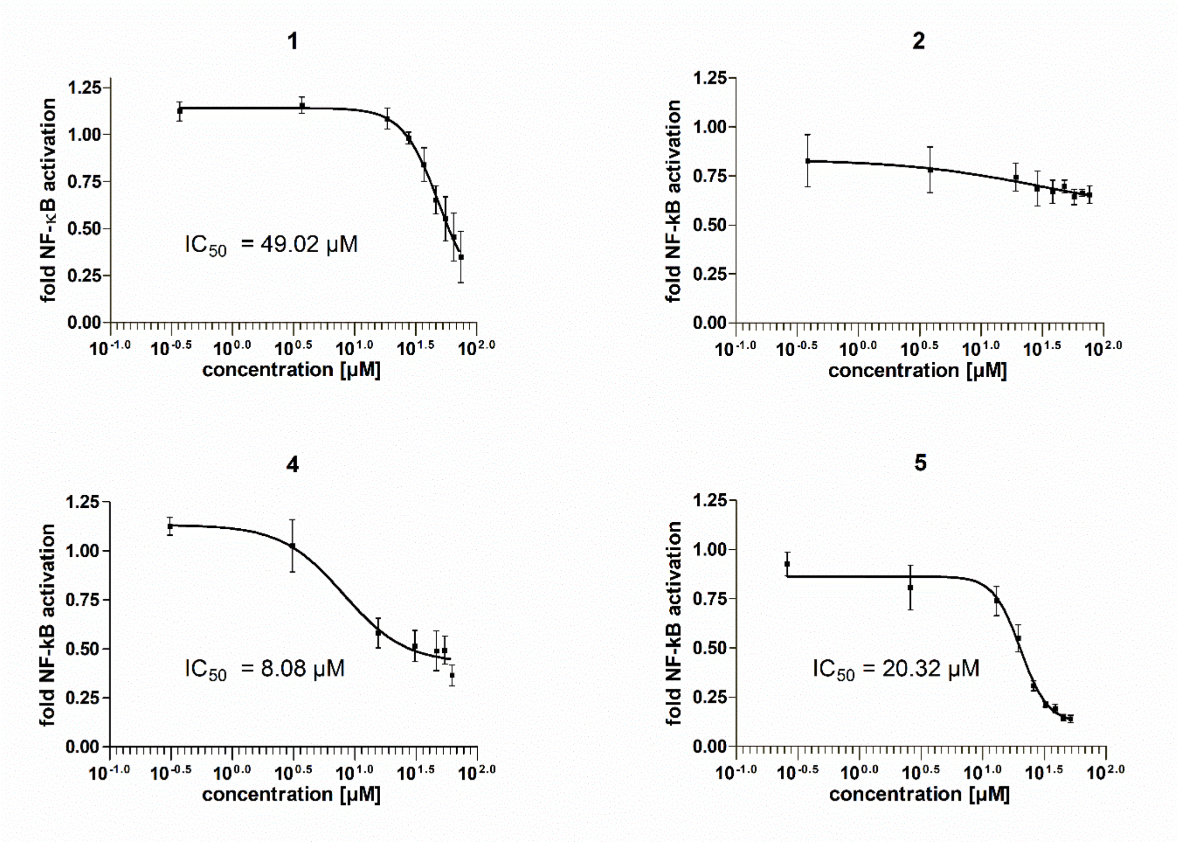

Figure S7. Concentration-response curves showing the positively correlated compounds **1**, **2**, **4,** and **5** assayed in a cell-based NF-κB-reporter gene assay at different concentrations. Shown are the residual NF-κB activities in TNF*α* (2 ng/mL)-activated HEK293-NF-κB-luc cells after treatment with the respective compounds. The luciferase signal derived from the NF-κB reporter was normalized to the CTG-fluorescence and expressed as fold change normalized to the TNF*α* signal. IC_{50} values were determined by non-linear regression with the sigmoidal dose response settings (variable slope) using GraphPad Prism 4.03 software.

Figure S8

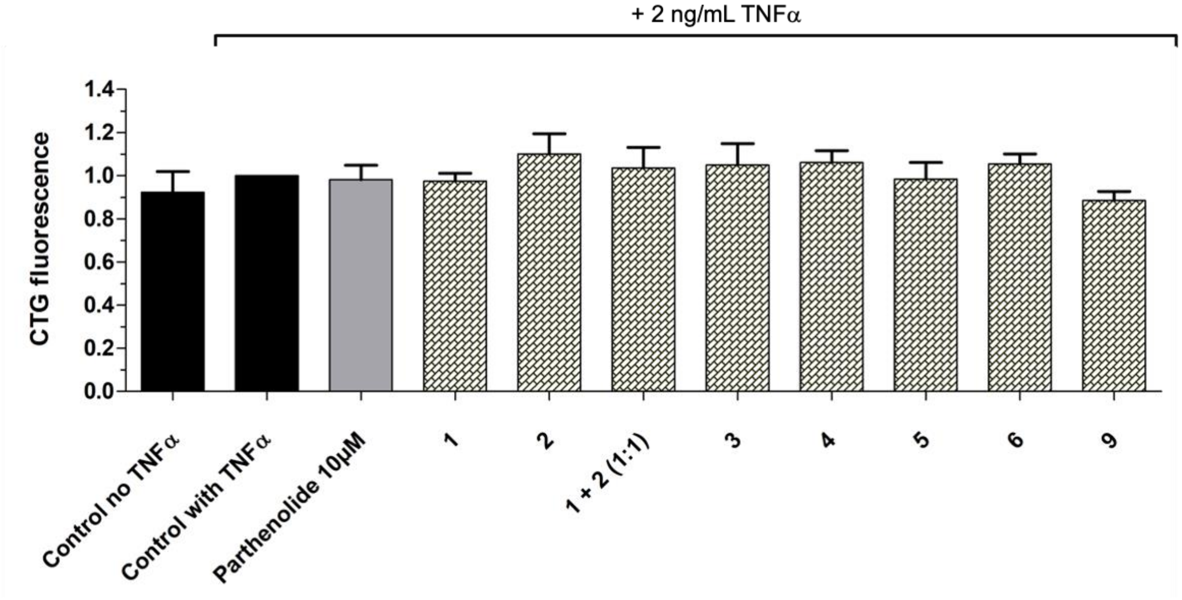

Figure S8. CTG fluorescence as a measure for cell viability after treatment with pure compounds **1**-**6**, **9** and a 1:1 mixture of **1** and **2** (all tested at 10 µg/mL) and the positive control parthenolide (10 µM) in TNFα (2 ng/mL)-activated HEK293-NFKB-luc cells; data are normalized to vehicle control (0.1% DMSO) with TNFα. Data represent mean ± SD. $n = 4$

Figure S9

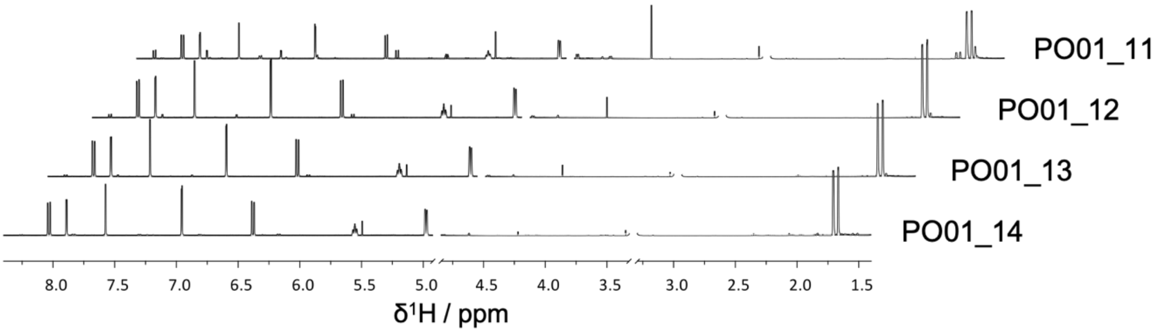

Figure S9. ^{1}H NMR data of package I (PO01_11-PO01_14).
